# Complete mitochondrial genome of *Aphlugiolopsis punctipennis* (Orthoptera: Tettigoniidae: Meconematinae)

**DOI:** 10.1128/mra.01206-25

**Published:** 2026-02-12

**Authors:** Rui Han, Tingting Yu, Xun Bian, Bin Zhang

**Affiliations:** 1 College of Life Sciences & Technology, Inner Mongolia Normal University 71203 , Hohhot, China; 2 Key Laboratory of Ecology of Rare and Endangered Species and Environmental Protection, (Guangxi Normal University), Ministry of Education 12388, Guilin, China; 3 Key Laboratory of Biodiversity conservation and Sustainable utilization for College and University of Inner Mongolia Autonomous Region, Hohhot, China; University of Maryland School of Medicine, Baltimore, Maryland, USA

**Keywords:** mitogenome, China, *Aphlugiolopsis*

## Abstract

We present the complete mitochondrial genome of *Aphlugiolopsis punctipennis* from China. The mitogenome of *A. punctipennis* is a double-stranded circular structure with a length of 18,008 bp and is AT rich (73%). It contains 13 protein-coding genes, 22 transfer RNA genes, 2 ribosomal RNAs, and a control region.

## ANNOUNCEMENT

The genus *Aphlugiolopsis* was established by Wang, Liu, and Li, with *Aphlugiolopsis punctipennis* as the type species (1, 2). So far, the genus is distributed only in southwestern China (1–3). The species *A. punctipennis* is distributed in Damingshan, Guangxi (4, 5). To date, knowledge of this specimen is limited to morphological character description. To enrich molecular data and facilitate phylogenetic studies of *A. punctipennis*, we sequenced and analyzed its complete mitochondrial genome.

In this study, a specimen of *A. punctipennis* was collected from the type locality Damingshan, Guangxi, China (23.503468 N, 108.439804 E), and deposited at Guangxi Normal University. Total DNA was extracted from the hind leg muscle using the TIANamp Genomic DNA Kit (TIANGEN), and high-throughput sequencing was subsequently performed by Beijing Berry Genomics Co., Ltd. The 150 bp paired-end library was constructed using the MGIEasy Kit (MGI) and sequenced on an Illumina NovaSeq 6000 (Illumina Inc.). After sequencing, the raw data were processed with fastp v0.20.0 (6) by trimming adapters and primers, filtering reads with Phred quality <Q5, and filtering reads with N base number >3. The newly generated sequence was assembled by NOVOPlasty v4.3.3 (7), with *Phlugiolopsis tuberculata* (NC_068779.1) as reference. Genome annotation was performed using the MITOS (v2.1.9) platform with the following parameter settings: Genome range = 14,000–18,000, K-mer = 39, Max memory = 16, and Extended log = 0; all other parameters were set to their default values (8–10). The mitogenome circle was mapped with Chloroplot v0.2.4 online site (11).

**Fig 1:**
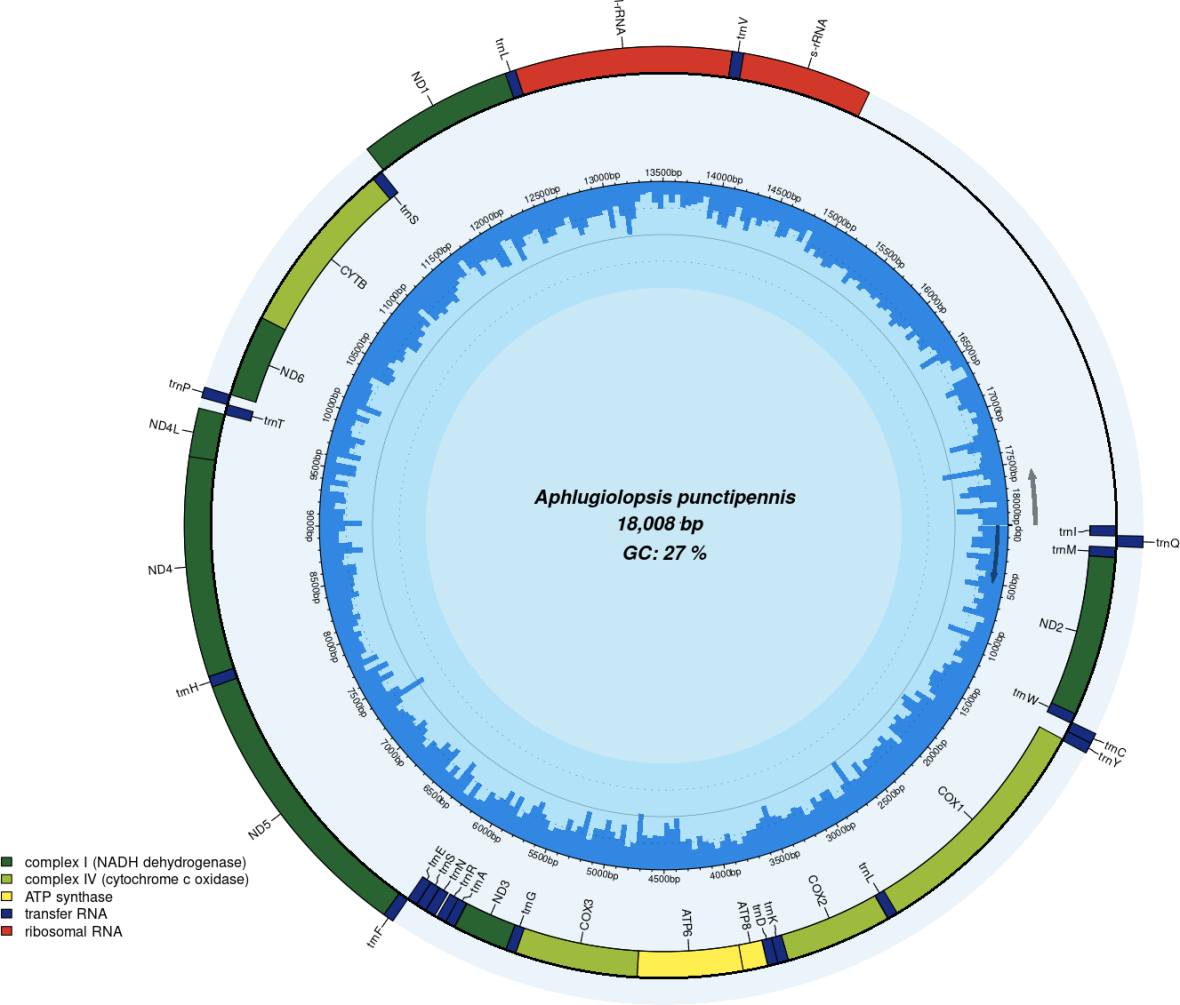
Circular map of the *Aphlugiolopsis punctipennis* mitochondrial genome: with a total length and GC content. Functional elements, including respiratory chain complex genes, tRNA genes, and rRNA genes, are distinguished by color coding, whereas the central peak graph illustrates the GC content distribution across the genome.

The complete mitogenome of *Aphlugiolopsis punctipennis* (PX412916.1) is a circular double-stranded structure with a length of 18,008 bp. The mitogenome is composed of a control region and 37 genes, including 13 PCGs, 22 tRNAs, and 2 rRNAs (16sRNA and 12sRNA), as summarized in Fig. 1. Table 1 shows the 13 PCGs, four type start codons ATG (cob, nad4, nad4L, cox3, atp6, cox2), ATT (nad5, nad3, atp8, cox1, nad2), TTG (nad1), and ATA (nad6), and three stop codons incomplete T (nad4, nad5, cox3, atp6, atp8, cox2), TAG (nad1, nad4L, cob, nad3), and TAA (nad6, cox1, nad2) in turn. The tRNA genes contain 22 sequences, with lengths ranging from 64 bp (trnR) to 72 bp (trnV). The rRNA genes comprise rrnS (790 bp) and rrnL (1,312 bp).

**TABLE 1:** Gene composition and annotation of *Aphlugiolopsis punctipennis*

Gene	Type	Start	End	Length (bp)	Start/stop codons	Intergenic region length (bp)	Direction
tRNA-Ile	tRNA	1	66	66	–^*a*^	0	Forward
tRNA-Gln	tRNA	63	132	70	–	−4	Reverse
tRNA-Met	tRNA	138	203	66	–	5	Forward
nad2	CDS	204	1232	1,029	ATT/TAA	0	Forward
tRNA-Trp	tRNA	1230	1296	67	–	−3	Forward
tRNA-Cys	tRNA	1288	1352	65	–	−9	Reverse
tRNA-Tyr	tRNA	1352	1417	66	–	−1	Reverse
cox1	CDS	1410	2954	1,545	ATT/TAA	−8	Forward
tRNA-Leu	tRNA	2949	3015	67	–	−6	Forward
cox2	CDS	3018	3702	685	ATG/T	2	Forward
tRNA-Lys	tRNA	3702	3772	71	–	−1	Forward
tRNA-Asp	tRNA	3771	3837	67	–	−2	Forward
atp8	CDS	3838	3997	160	ATT/T	0	Forward
atp6	CDS	3996	4671	676	ATG/T	−2	Forward
cox3	CDS	4672	5458	787	ATG/T	0	Forward
tRNA-Gly	tRNA	5458	5523	66	–	−1	Forward
nad3	CDS	5524	5877	354	ATT/TAG	0	Forward
tRNA-Ala	tRNA	5881	5945	65	–	3	Forward
tRNA-Arg	tRNA	5944	6007	64	–	−2	Forward
tRNA-Asn	tRNA	6023	6089	67	–	15	Forward
tRNA-Ser	tRNA	6091	6157	67	–	1	Forward
tRNA-Glu	tRNA	6158	6225	68	–	0	Forward
tRNA-Phe	tRNA	6223	6288	66	–	−3	Reverse
nad5	CDS	6289	8020	1,732	ATT/T	0	Reverse
tRNA-His	tRNA	8020	8085	66	–	−1	Reverse
nad4	CDS	8086	9424	1,339	ATG/T	0	Reverse
nad4l	CDS	9418	9714	297	ATG/TAA	−7	Reverse
tRNA-Thr	tRNA	9715	9780	66	–	0	Forward
tRNA-Pro	tRNA	9779	9844	66	–	−2	Reverse
nad6	CDS	9846	10373	528	ATA/TAA	1	Forward
cob	CDS	10373	11509	1,137	ATG/TAG	−1	Forward
tRNA-Ser	tRNA	11507	11576	70	–	−3	Forward
nad1	CDS	11593	12540	948	TTG/TAG	16	Reverse
tRNA-Leu	tRNA	12540	12604	65	–	−1	Reverse
16sRNA	rRNA	12604	13914	1,311	–	−1	Reverse
tRNA-Val	tRNA	13917	13988	72	–	2	Reverse
12sRNA	rRNA	13987	14776	790	–	−2	Reverse

^*a*^ –, not applicable.

## Data Availability

The complete mitochondrial genome sequence of *Aphlugiolopsis punctipennis* is available in GenBank under the accession number PX412916.1. The corresponding BioProject, BioSample, and SRA numbers are PRJNA1335606, SAMN52016402, and SRR35985027, respectively.
